# Supplementary material for: Biomaterial-Mediated Protein Expression Induced by Peptide-mRNA Nanoparticles Embedded in Lyophilized Collagen Scaffolds
Source: Pharmaceutics. 2022 Aug 2;14(8):1619. doi: 10.3390/pharmaceutics14081619 (PMC9414905; doi:10.3390/pharmaceutics14081619)
Supplement: Supplementary file 1 [file pharmaceutics-14-01619-s001.zip › pharmaceutics-1825698-supplementary.pdf]

pharmaceutics

MDPI

Supplementary Materials: Biomaterial-Mediated Protein Expression Induced by Peptide-mRNA Nanoparticles Embedded in Lyophilized Collagen Scaffolds

Rik Oude Egberink, Helen M. Zegelaar, Najoua El Boujnouni, Elly M. M. Versteeg, Willeke F. Daamen and Roland Brock

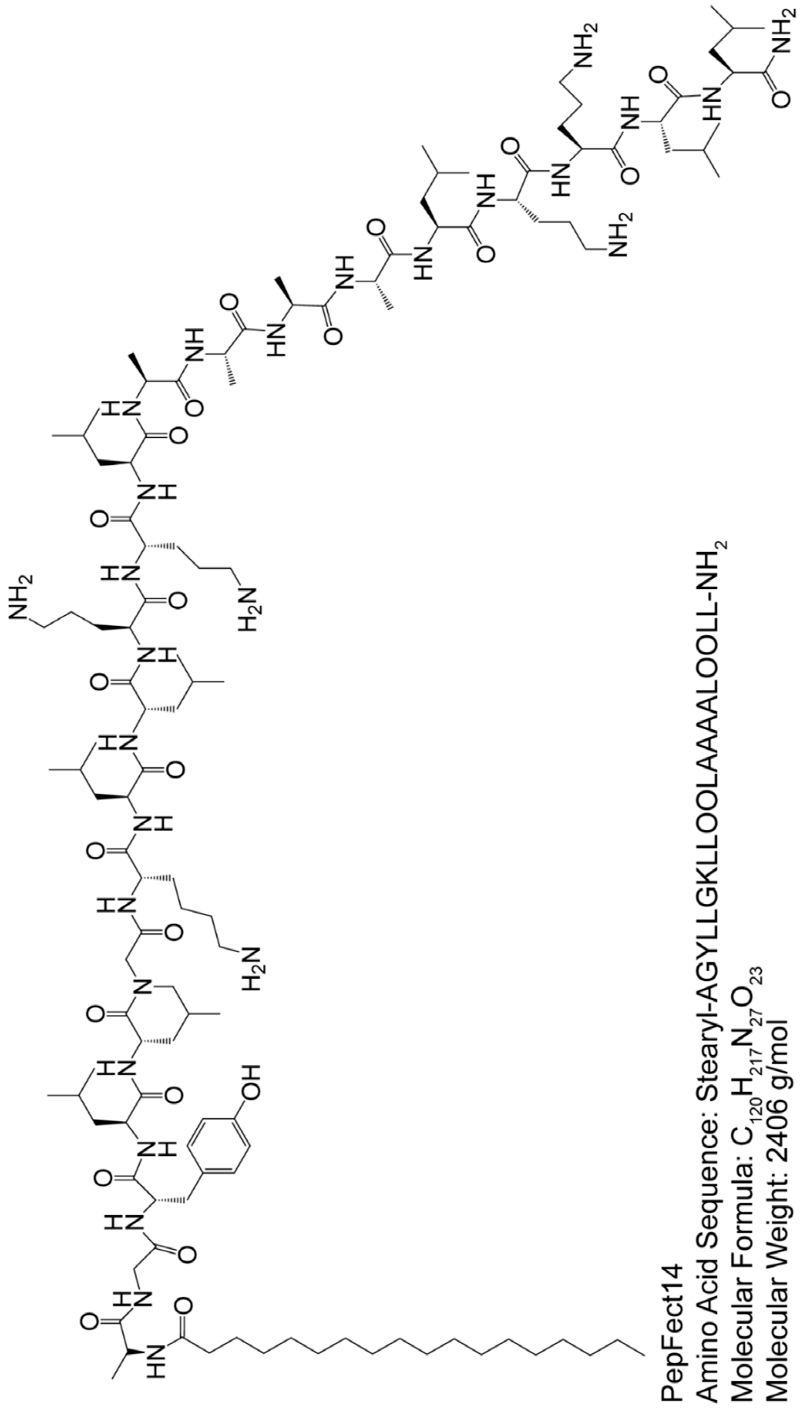

Figure S1. The chemical structure of the amphipathic cationic cell-penetrating peptide PepFect14. The chemical structure was drawn with ChemDraw version 21.0.0 (Perkin Elmer, Waltham, MA, USA).

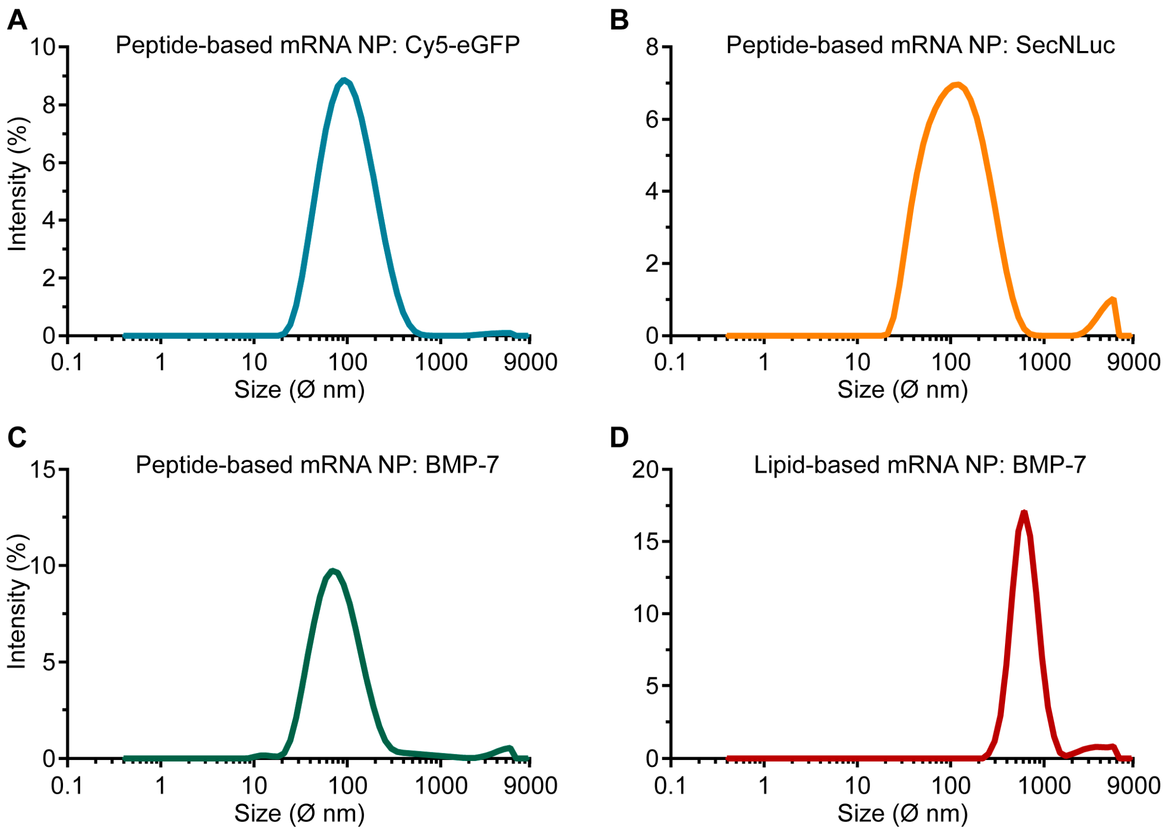

Figure S2. Characterization of peptide-based (PF14) and lipid-based (LMM) mRNA transfection complexes. (**A**) Intensity-based particle size distribution of PepFect14 formulated with Cy5-eGFP mRNA. (**B**) Intensity-based particle size distribution PepFect14 formulated with SecNLuc mRNA. (**C**) Intensity-based particle size distribution PepFect14 formulated with BMP-7 mRNA. (**D**) Intensity-based particle size distribution Lipofectamine MessengerMAX formulated with BMP-7 mRNA.

Table S1. Quantitative results of DLS measurements. Averages and standard deviations reflect four repeated measurements. PdI: polydispersity index.

Sample	Z-Average (Ø nm)	PdI	Attenuator	Intercept	Derived Counts
20 μM PF14 + Cy-5 eGFP	84.86 ± 1.59	0.250 ± 0.047	7	0.146 ± 0.011	44,188 ± 3,029
50 μM PF14 + SecNLuc	95.00 ± 4.50	0.376 ± 0.008	9	0.924 ± 0.004	3,052 ± 17
50 μM PF14 + BMP-7	69.49 ± 3.21	0.258 ± 0.014	9	0.940 ± 0.004	3,058 ± 27
LMM + BMP-7	631.3 ± 12.4	0.249 ± 0.056	8	0.917 ± 0.004	9,362 ± 154

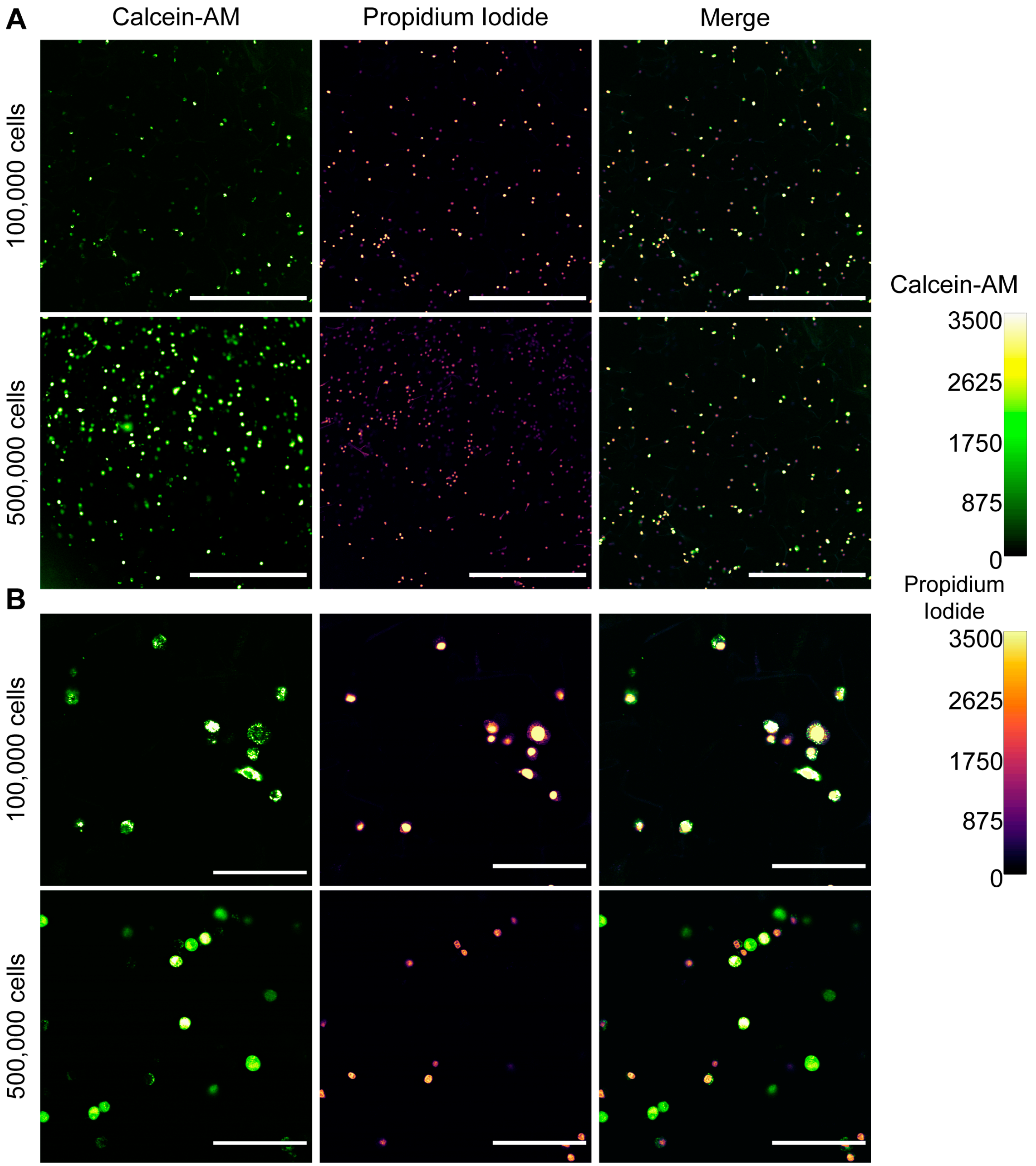

Figure S3: Dependence of cell viability and spreading in 3D collagen scaffolds on seeding density for C2C12 cells. **(A)** Low magnification overview of C2C12 viability 48 h post-seeding; **(B)** high magnification zoom-in. Calcein-AM intensities to assess cell viability and PI staining are visualized by false color look-up tables green hot and mpl-inferno, respectively (right). Brightness and contrast were equally adjusted across conditions. Scale bars in panel A represent 500 µm, scale bars in panel B represent 100 µm.

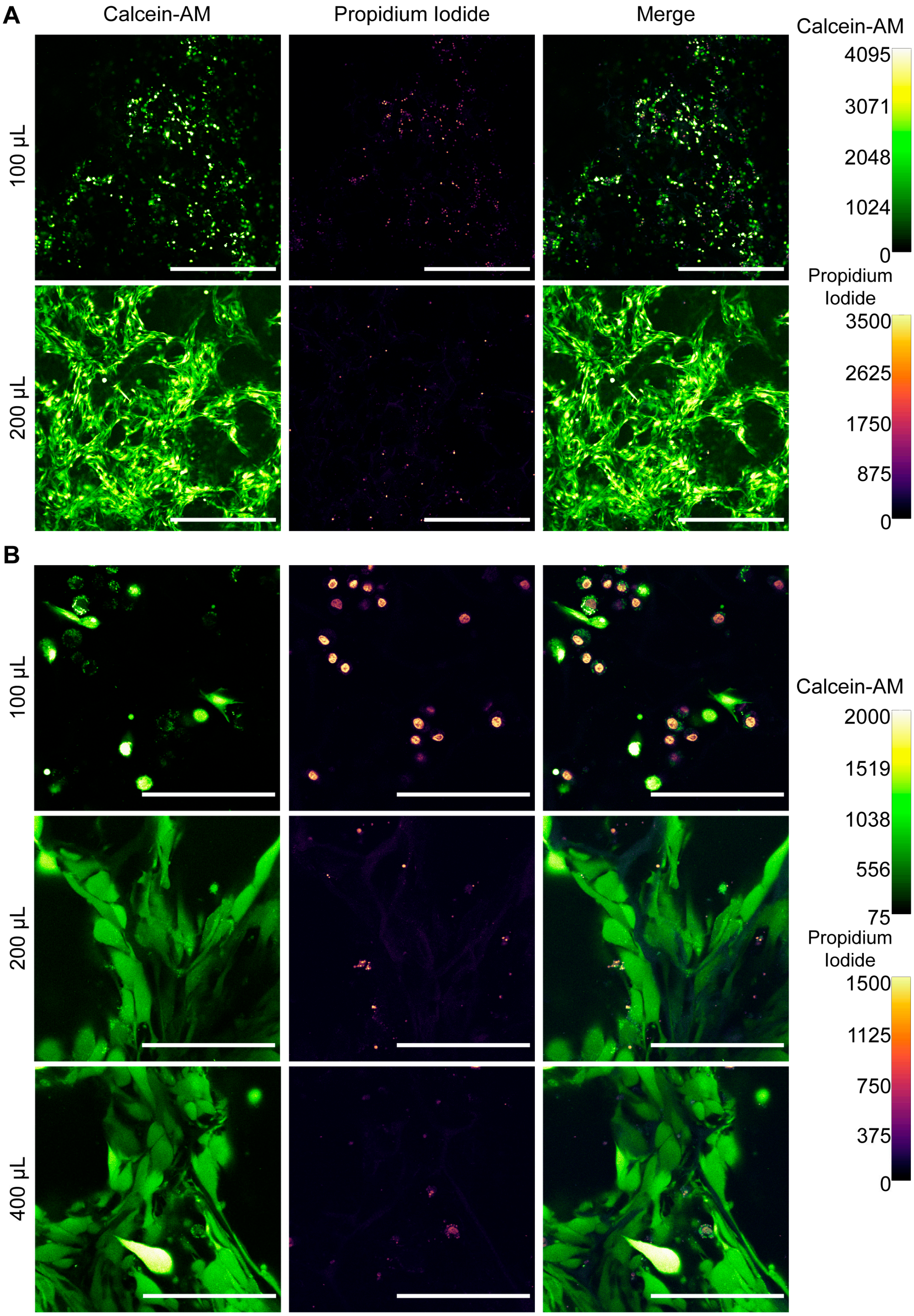

Figure S4. The effects of pre-lyophilization volume on the presence of viable C2C12 cells throughout a 3D collagen scaffold. **(A)** Low magnification overview of C2C12 viability 24 h post-seeding at different seeding volumes. **(B)** High magnification overview of C2C12 viability viability 24 h post-seeding at different seeding volumes. The green hot LUT depicts calcein-AM intensities, while the mpl-inferno LUT depicts the PI intensity. Brightness and contrast were equally adjusted across conditions per panel according to the calibration bar. Scale bars in panel A represent 500 µm, scale bars in panel B represent 100 µm.

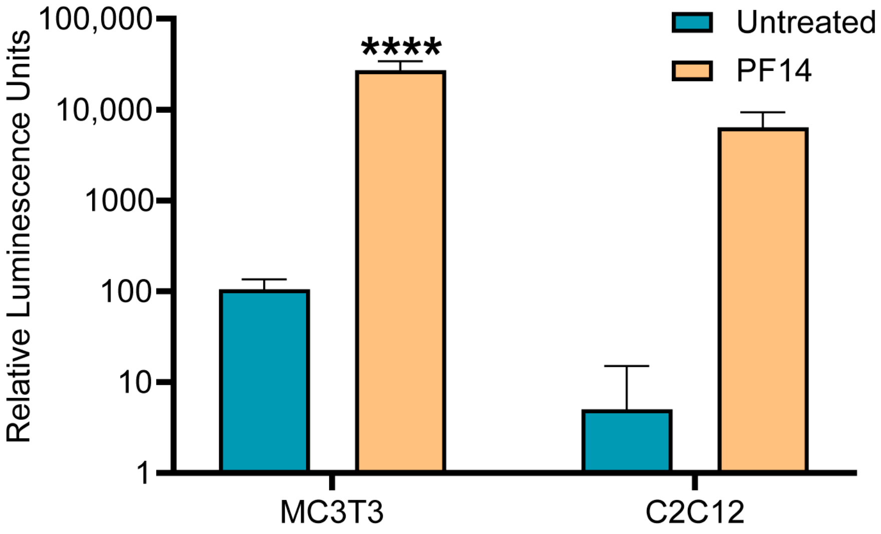

Figure S5. Comparison of transfection efficiencies between MC3T3 and C2C12 cells, transfected with 5 μM (536 ng mRNA/scaffold) PF14-SecNLuc mRNA nanoparticles embedded in 3D collagen scaffolds. Values represent the mean + SD of three biological replicates, of total luciferase production (sum of secreted and sequestered luciferase). ****:P ≤ 0.0001

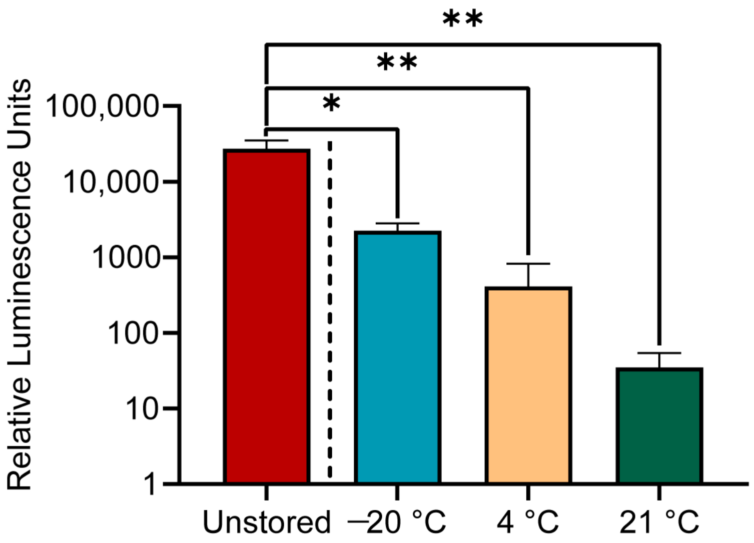

Figure S6. Comparison of MC3T3 transfection efficiencies of PF14 mRNA-loaded scaffolds (536 ng mRNA/scaffold) stored at different temperatures for 14 days. Data represent the mean + SD of three biological replicates of both the secreted and sequestered nanoluciferase. The dashed line separates data that was acquired in separate experiments. Statistically significant differences were determined by means of a one-way ANOVA. *:P ≤ 0.05, **:P ≤ 0.01.
